# Probing Neural Compensation in Rehabilitation of Acute Ischemic Stroke with Lesion Network Similarity Using Resting State Functional MRI

**DOI:** 10.3390/brainsci15090964

**Published:** 2025-09-04

**Authors:** Shanhua Han, Quan Tao, Boyu Zhang, Yifan Lv, Zhihao Li, Yu Luo

**Affiliations:** 1 Department of Radiology, Shanghai Fourth People’s Hospital, School of Medicine, Tongji University, Shanghai 200434, China; sena-han@hotmail.com (S.H.); 2205093@tongji.edu.cn (Q.T.); boyuzhang@fudan.edu.cn (B.Z.); lv777evvan@tongji.edu.cn (Y.L.); 2 Department of Psychiatry and Behavioral Sciences, Emory University, Atlanta, GA 30322, USA

**Keywords:** acute ischemic stroke, rehabilitation, resting state functional MRI, Lesion network analysis, compensation

## Abstract

**Background/Objectives**: Neural compensation, in which healthy brain regions take over functions lost due to lesions, is a potential biomarker for functional recovery after stroke. However, previous neuroimaging studies often speculated on neural compensation simply based on greater measures in patients (compared to healthy controls) without demonstrating a more direct link between these measures and the functional recovery. Because taking over the function of a lesion region means taking on a similar role as that lesion region in its functional network, the present study attempted to explore neural compensation based on the similarity of functional connectivity (FC) patterns between a healthy regions and lesion regions. **Methods**: Seventeen stroke patients (13M4F, 63.2 ± 9.1 y.o.) underwent three resting-state functional MRI (rs-fMRI) sessions during rehabilitation. FC patterns of their lesion regions were derived by lesion network analysis; and these patterns were correlated with healthy FC patterns derived from each brain voxel of 51 healthy subjects (32M19F, 61.0 ± 14.3 y.o.) for the assessment of pattern similarity. **Results**: We identified five healthy regions showing decreasing FC similarity (29–54%, all corrected *p* < 0.05, effect size η^2^: 0.10–0.20) to the lesion network over time. These decreasing similarities were associated with increasing behavioral scores on activities of daily living (ADL, *p* < 0.001, η^2^ = 0.90), suggesting greater neural compensation at early-stage post-stroke and reduced compensation toward the end of effective rehabilitation. **Conclusions**: Besides direct FC measures, the present results propose an alternative biomarker of neural compensation in functional recovery from stroke. For sensorimotor recoveries like ADL, this biomarker could be more sensitive than direct measures of lesion connectivity in the motor network.

## 1. Introduction

Stroke, acute ischemic in particular, is a critical medical emergency characterized by a sudden reduction in blood flow to the brain, leading to the death of brain cells [1,2]. It manifests through a range of acute symptoms, including sudden-onset numbness or weakness in the limbs, facial droop, difficulties in speaking or understanding, confusion, impaired balance or coordination, and vision loss [1,3]. The impacts of ischemic stroke extend profoundly into an individual’s daily life, significantly impairing their ability to perform activities of daily living (ADL). Among the various disabilities resulting from stroke, motor impairment is the most prevalent, restricting muscle movement and overall mobility [4,5].

Rehabilitation programs for stroke often include physical therapy, occupational therapy, biofeedback therapy, speech therapy, and, when necessary, psychological counseling [3,6,7,8]. Evidence demonstrates that these rehabilitation efforts are effective in improving long-term ADL, underscoring their efficacy in post-stroke recovery [8]. However, behavioral outcomes often exhibit large variations, hence subjective evaluations from therapists are often involved in their rehabilitative assessments and decisions. For these therapists, objective biomarkers based on functional neuroimaging are desirable as an additional/alternative evaluation.

One biomarker often discussed in the literature is neural compensation [9]. It broadly refers to the recruitment or reorganization of healthy brain regions to take over functions lost due to the lesion’s damage [10]. However, many previous neural imaging studies simply address neural compensation in a post hoc and speculative manner [11,12,13,14]. Compared with a healthy/control group, if patients show a decreased imaging measure (e.g., fractional anisotropy or functional activation), then that measure is often interpreted as an impairment or deficit; but if patients show an increased measure, then that measure is often speculated to indicate compensation. Such speculations, though straightforward, still require further examination. Specifically, when discussing neural compensation in stroke rehabilitation, we need to at least examine two aspects. First, if a healthy brain region is taking over the function of a lesion region, then this healthy region should manifest some functional characteristics of that lesion region. Second, if a compensation measure represents the neural mechanism of some rehabilitation (biomarker), then this compensation measure should manifest an association with behavioral recovery. However, instead of a post hoc speculation simply based on increased patient measures than controls, explorations for compensation evidence in these directions are still lacking.

**Figure 1:**
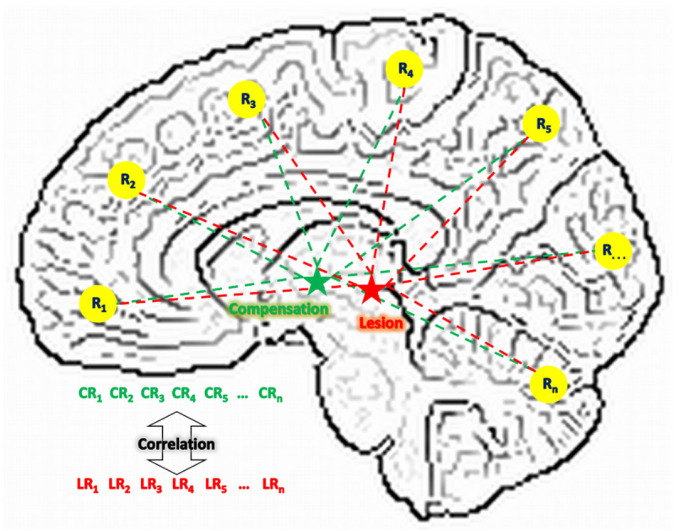
The compensational hypothesis of this study. L: lesion; C: compensation; R: region; LR: connectivity between a lesion region and a regular region; CR: connectivity between a compensation region and a regular region.

Lesion network (LN) analysis has the potential for providing demonstrations on these two aspects. With resting-state functional MRI (rs-fMRI) data, this approach estimates the connectivity pattern of a lesion region based on that of the same region in healthy brains [15,16]. As shown in Figure 1, suppose a lesion (L) region was connected to n other regions (R) when it was healthy; its connectivity pattern can be denoted as a vector of $V_{LR}\;=\;[{LR}_{1},{LR}_{2},\;\ldots ,{LR}_{n}]$. Because a lesion region is not functioning, this vector cannot be directly assessed from the patient. But after spatially mapping this lesion region to the same location in a healthy brain, this vector is then assessable from a group of healthy subjects through the analysis of functional connectivity. Meanwhile, if a compensation (C) region exists, its connectivity pattern with the same n regions can be denoted as another vector of $V_{CR}\;=\;[{CR}_{1},{CR}_{2},\;\ldots ,{CR}_{n}]$. Because a compensation region is healthy and functioning, this vector can be derived directly from the patient. Since compensation means partially replacing a lesion region’s original function, we hypothesize that the level of this compensation can be represented by the similarity between these two vectors of $V_{LR}$ and $V_{CR}$. In other words, LN analysis may address neural compensation from the perspective of network resemblance. The more similarity between $V_{LR}$ and $V_{CR}$, the compensation region is acting more like the lesion region in the functional network, hence replacing to a greater extent the lesion region’s role. Therefore, by searching in a patient’s brain with voxel-wise-derived $V_{CR}$ and comparing with the $V_{LR}$ derived from a group of healthy subjects, we can identify compensation regions based on high vector similarity and examine the change of this compensation across time.

With such an approach, this study aims to examine neural compensation in stroke by exploring compensatory areas through analyses of lesion network similarity. With rs-fMRI data acquired from stroke patients in a rehabilitation program, we investigated whether similar network connectivity patterns between lesion regions and compensatory regions can be identified and how these patterns evolve throughout the rehabilitation process. We hypothesized that lesions associated with stroke symptoms may share their connectivity profiles with compensating regions, and these shared connectivity profiles may change as a function of the rehabilitation procedure.

## 2. Materials and Methods

### 2.1. Subjects

This study included 19 patients diagnosed with acute ischemic stroke who were admitted to Shanghai Fourth People’s Hospital (Shanghai, China). Their enrollment was approved by the hospital’s medical research ethics committee with informed consent. Clinical information collected from each stroke patient included age, gender, days from symptom onset to MRI, details of comprehensive rehabilitation therapy (encompassing physical therapy, occupational therapy, speech therapy, and psychological counseling when necessary) [2], activities of daily living (ADL) scores [17], and relevant clinical history.

Data of rs-fMRI were acquired from all patients through three imaging sessions, at the beginning (about 1 week after the onset of stroke), in the middle (~3 months), and at the end (~6 months), respective, of their rehabilitation program. Because the rehabilitative effect needs to be assessed with data from at least 2 imaging sessions, 2 patients (both male, age 65 and 66, ADL 25/50/80 and 40/70/95) were excluded for having usable (criteria described below) rs-fMRI data in only 1 session.

To derive the lesion networks (LN) of these patients, healthy subjects’ rs-fMRI data were acquired from two sources, one being local and the other being remote to the patients. The local source included 20 subjects (with informed consent) in a similar age range and from the same local resident community as the patients; and they were scanned once with the same scanner and identical imaging parameters. The remote source included 946 subjects from the “1000 Functional Connectomes Projects” (https://www.nitrc.org/projects/fcon_1000, accessed on 16 September 2019), which is a publicly released rs-fMRI dataset with global contributors.

To control the heterogeneity in the healthy subjects to obtain a LN more specific to the present stroke patients, the local and remote sources of healthy subjects were pooled together and screened with a procedure of propensity score matching [18]. Specifically, each stroke patient was matched with 3 healthy controls based on age, gender, and max head motion averaged across the 3 imaging scans. Based on the propensity score, the top 3 matching subjects from the healthy pools were picked for each stroke patient. This procedure yielded 51 healthy subjects (15 local + 36 remote) and their demographic comparisons with the patients are shown in Table 1.

### 2.2. Imaging Parameters

All images were acquired on a Siemens Avanto 1.5T MR system (Siemens, Erlangen, Germany) at Shanghai Fourth People’s Hospital. Whole brain rs-fMRI acquired using an echo-planar imaging (EPI) sequence: 21 axial slices, thickness = 3.4 mm, gap = 2.6 mm, matrix = 64 × 64, repetition time (TR) = 3000 ms, measurement = 130, echo time (TE) = 30 ms, flip angle = 90°, and field of view (FOV) = 220 mm × 220 mm. T1-weighted images were obtained in a sagittal orientation with a magnetization-prepared rapid gradient echo (MPRAGE) sequence: 176 slices per slab, thickness = 1 mm, no gap, TR = 2500 ms, TE = 2.42 ms, FOV = 256 mm × 256 mm, flip angle = 15°, and matrix = 256 × 256. During rs-fMRI, participants were instructed to remain awake and relaxed with their eyes closed without thinking about anything in particular. The total scan time, including anatomical and functional scans, was 13 min.

### 2.3. Imaging Data Analysis

The present data analyses were carried out using the software package AFNI (linux_openmp_64, November 2022, afni.nimh.nih.gov). The image preprocessing pipeline included 9 steps of (i) outlier detection (AFNI’s 3dToutcount), (ii) signal despiking (3dDespike), (iii) slice timing correction (3dTshift), (iv) volume registration (3dvolreg), (v) anatomy-to-function registration (align_epi_anat.py), (vi) nuisance signal (head motion and derivatives, white matter, cerebral spinal fluid) regression (3dTproject), (vii) bandpass (0.009–0.08 Hz) filtering (3dFourier), (viii) spatial smoothing (3dBlurInMask, FWHM = 5 mm), and (ix) spatial normalization into the MNI space (3dQwarp). At the end of this preprocessing pipeline, rs-fMRI scans were screened by 3 criteria for inclusion in the subsequent analysis [19]: (1) after excluding timepoints with signal outliers and excessive head motion, the remaining time course should still be longer than 4.7 min; (2) maximum pair-wise head motion should be less than 2 mm throughout the scan; and (3) the proportion of timepoints excluded (aka. censored/scrubbed) should be less than 20%.

**Figure 2:**
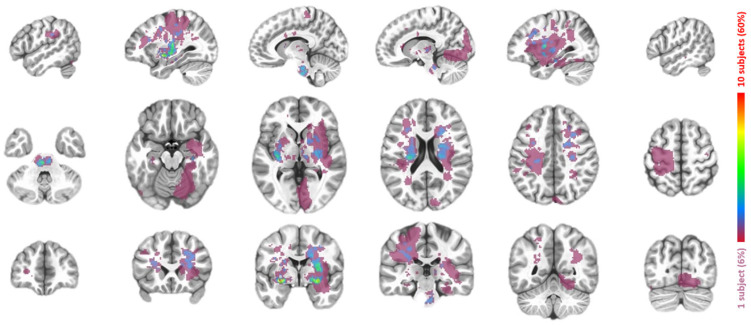
Superimposed lesion regions shown in different slices in sagittal (top row), axial (middle row), and coronal (bottom row) views. The color coding indicates how many patients shared a lesion voxel.

**Figure 3:**
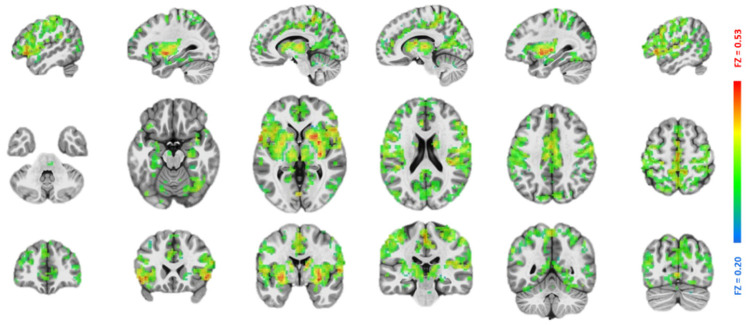
The lesion network derived from the 51 healthy subjects. (*p* < 0.001/voxel plus 216 mm^3^ cluster, *p* < 0.05 corrected).

After data preprocessing, the analysis continued by deriving the LN for each patient using their lesion regions (Figure 2) manually traced by a radiologist (author S.H.). Specifically, each patient’s lesion generated 51 (51 matched healthy subjects) functional connectivity (FC) maps with each voxel containing a Fisher’s Z value. There FC maps were generated by simply correlating the mean signal of the lesion mask with all voxels in the brain. For a total of 17 patients, this procedure generated 867 FC maps, with each map associated with the (i) age, (ii) gender, (iii) max motion, and (iv) imaging site of a particular healthy subject, as well as (v) the lesion region of a particular patient. To derive the LN at the group level, these 867 maps were merged through multivariate modeling (AFNI’s 3dMVM) with variations associated with (i) to (v) removed. The voxel-wise intercept from this modeling was then extracted as the LN (Figure 3) for the subsequent analysis.

Using this healthy-subject-derived-LN as the template, the similarity (Pearson correlation and then Fisher’s Z transformation) between this template and each voxel’s connectivity pattern was calculated for each patient’s imaging visit; and this similarity score was assigned to the corresponding voxel. In other words, at each imaging visit of each patient, a map of functional compensation was derived, with each voxel’s value representing functional compensation of that voxel; and this compensation was measured as the similarity between that voxel’s connectivity pattern and the LN. Subsequently, these compensation maps were submitted to a voxel-wise linear mixed effect (LME) modeling [20] of

(1) Similarity ~ ADL+Age+Gender+MaxHeadMotion

Here, the independent variables on the right-hand side were individual values taken at each imaging visit with the MaxHeadMotion being the maximum pairwise distance (Euclidean norm) derived from the 6-dimension assessment of head motion. The interested outcome of this LME model was brain regions showing a significant ADL effect. Once these regions were identified, for result visualization, group level connectivity maps were generated for each of these identified regions with an LME modeling of

(2) Connectivity~ADL+Age+Gender+MaxHeadMotion

Model (1) and (2) are mostly identical, with the only difference being the dependent variable on the left-hand side. With model (2), we derived connectivity maps at 3 ADL levels of 47, 65, and 83, respectively, with the variations associated with age, gender, and head motion removed. Note that the model expression (2) does not mean a neurological dependence of functional connectivity on Al, it simply models the association between these two variables. Neurologically, the behavioral measure of ADL should depend on the functional neural network.

All the present voxel-wise statistics were corrected for multiple comparison with probability of false-positive clusters estimated by Monte Carlo simulations implemented in AFNI’s 3dClustSim function. Taking into account the spatial smoothness of the noise and non-Gaussian spatial autocorrelation, this method combines a voxel-wise threshold with a cluster-size threshold to control the family-wise error rate [21].

## 3. Results

### 3.1. Participants and Non-Imaging Data

A total of 19 right-handed patients with acute stroke and 51 healthy subjects were initially enrolled. After excluding 2 patients with insufficient fMRI data (i.e., only one usable imaging session), 17 stroke patients (13 males, 4 females; mean age 63.2 ± 9.1 years) and 51 healthy subjects (32 males, 19 females; mean age 61.0 ± 14.3 years) remained in the final analysis. Each stroke patient underwent three fMRI sessions at approximately 2.0, 104.6, and 210.3 days post-stroke onset (Table 1). Notably, their mean ADL scores increased from 32.2 ± 6.3 at the 1st imaging visit to 75.3 ± 18.2 at the 2nd visit, and to 88.4 ± 16.8 at the 3rd visit, reflecting functional recovery during the rehabilitation program.

### 3.2. Lesion Network Mapping

All lesions in different locations of the 17 patients were overlaid and visualized in Figure 2. As shown in Figure 3, the associated LN extended broadly into both cortical and subcortical regions. The most noticeable region in this LN was the thalamus. As a sanity check, because of its role as a relay station for sensorimotor signals, the salient thalamus involvement in the LN corresponds well with the ADL deficits in the present patients.

### 3.3. Identification of Compensatory Regions

**Figure 4:**
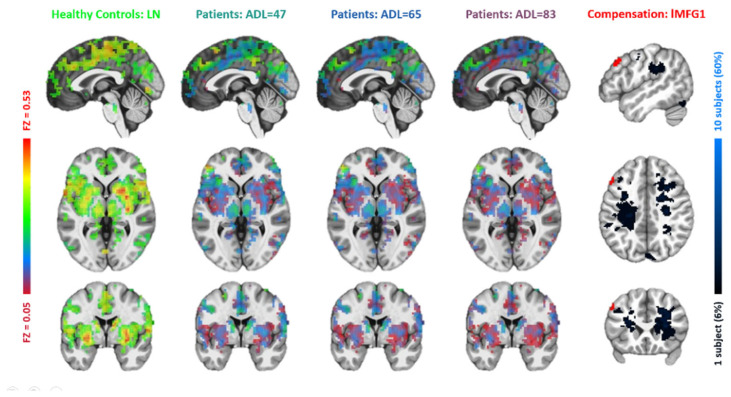
Comparisons between the lesion network (far-left column) with functional connectivity maps of the first left middle frontal gyrus (lMFG1) at three ADL levels of 47 (middle left), 65 (middle), and 83 (middle right), respectively. In the far-right column, the lMFG1′s position (red) is compared to the manually traced lesion regions (black-blue).

**Figure 5:**
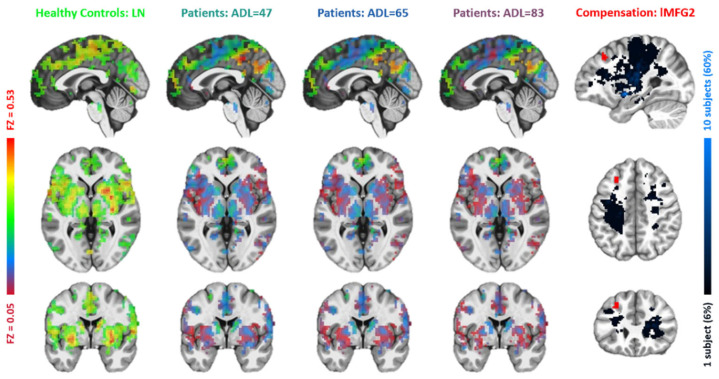
Comparisons between the lesion network with functional connectivity maps of the second left middle frontal gyrus (lMFG2). The figure layout and color coding are the same as in Figure 4.

**Figure 6:**
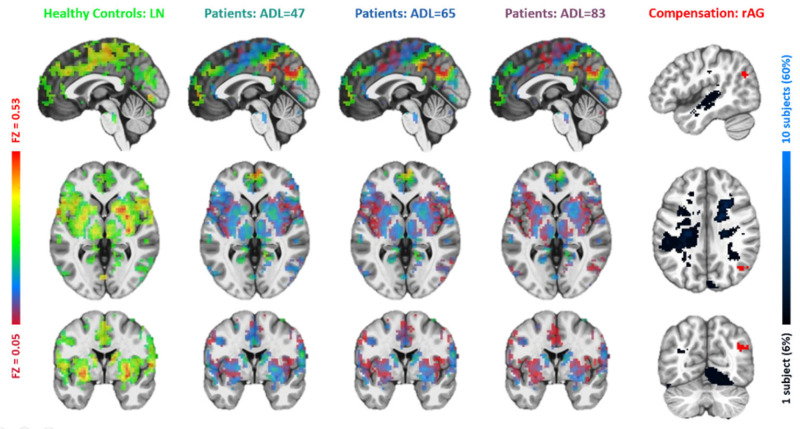
Comparisons between the lesion network with functional connectivity maps of the right angular gyrus (r_AngularGyrus). The figure layout and color coding are the same as in Figure 4.

**Figure 7:**
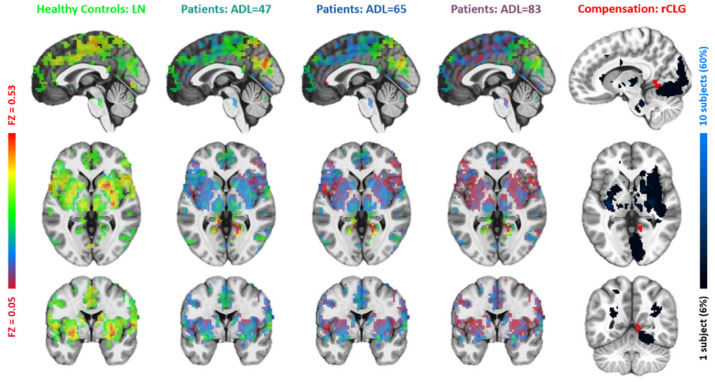
Comparisons between the lesion network with functional connectivity maps of the right culmen/lingual gyrus (r_Culmen/LingualGyrus). The figure layout and color coding are the same as in Figure 4.

**Figure 8:**
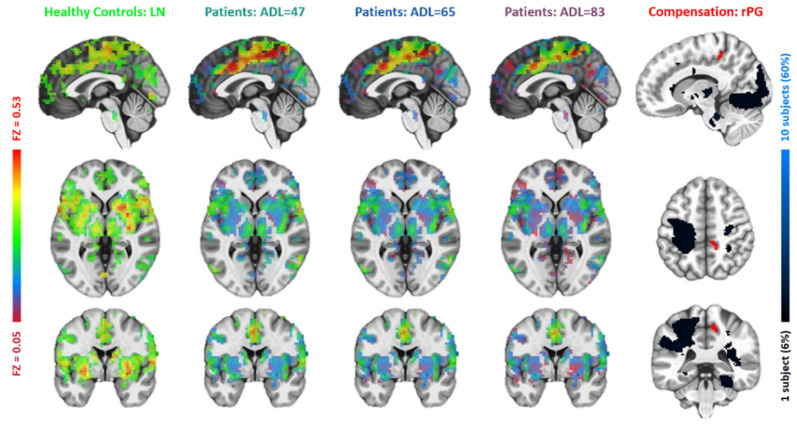
Comparisons between the lesion network with functional connectivity maps of the right paracentral gyrus (r_ParacentralGyrus). The figure layout and color coding are the same as in Figure 4.

**Figure 9:**
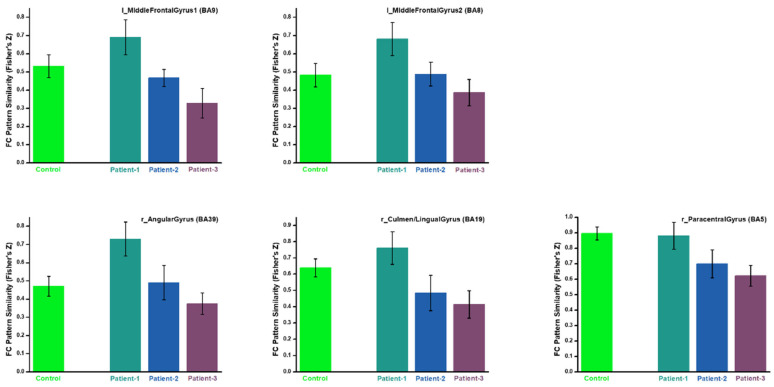
FC pattern similarity (to LN) of different regions of compensation. “Patient-1,2,3” means the three imaging visits of the patients. The green bar of “Control” represents the same FC pattern similarity when using the compensation regions as seeds on healthy subjects for deriving FC maps.

Based on the resemblance of functional connectivity (FC) profiles between healthy regions and lesion regions, comparing the LN with longitudinal patient data at ~1 week, ~3 months, and ~6 months post-stroke, the LME model (Equation (1)) identified five regions exhibiting a gradual decrease in such resemblance over time (Figure 4, Figure 5, Figure 6, Figure 7 and Figure 8). These regions (Table 2) included the left middle frontal gyrus (BA9/8), right angular gyrus (BA39), right culmen or lingual gyrus (BA19), and right paracentral gyrus (BA5). As patients showed recovery at each imaging visit, the decreasing similarity between these regions’ connectivity profiles and the LN (Figure 9) suggested stronger compensation at the beginning of stroke, which then gradually faded towards the end of the rehabilitation program. Also of notice are the locations of these five regions. As shown in the far-right columns of Figure 4, Figure 5, Figure 6, Figure 7 and Figure 8, these five compensation regions all resided beyond the lesion regions, consistent with the intuition that lesion regions should be compensated by healthy regions.

### 3.4. Lesion Connectivity with Traditional Motor Regions

**Figure 10:**
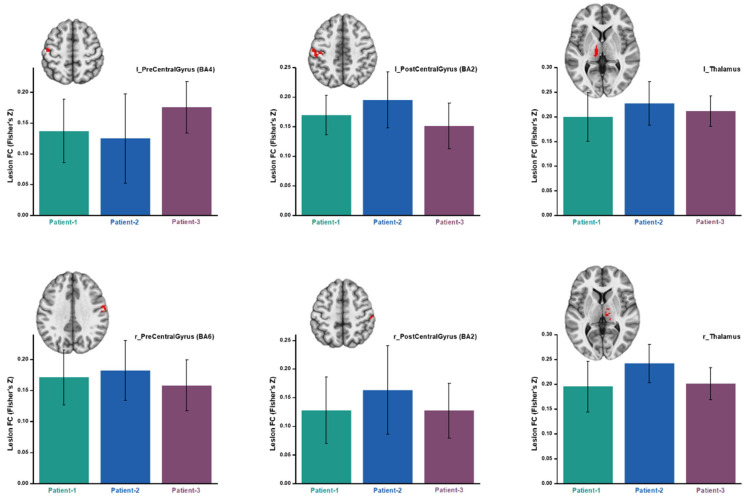
Changes in functional connectivity between the lesion and the six core regions (the red blobs) in the LN. “Patient-1,2,3” means the three imaging visits of the patients.

While the five identified healthy regions could be considered potential biomarkers of the rehabilitation, an extensive question one may ask is whether direct/simple functional connectivity, rather than the similarity of connectivity patterns, could also be derived as biomarkers of rehabilitation in the present patients. To explore in this direction, we applied a stringent “activation” threshold of *p* < 10^−5^ on the LN and picked six regions, the left/right precentral gyrus (BA4/BA6), left/right postcentral gyrus (BA2), and left/right thalamus (Figure 10), as the targets, for examining their direct FC with the lesion region. These six regions are tightly associated with sensorimotor functions [22,23,24], hence are likely to show direct FC changes, if there are any, in the present context of ADL recovery. However, as shown in Figure 10, these core sensorimotor areas did not exhibit significant (all *p* > 0.3) longitudinal changes in FC with the lesion region.

## 4. Discussion

The present study sought to clarify how network-level compensation supports the improvement of ADL during stroke rehabilitation. By analyzing rs-fMRI data and employing lesion network (LN) mapping, we identified five remote cortical regions (left middle frontal gyrus, right angular gyrus, right culmen/lingual gyrus, right paracentral gyrus) whose functional connectivity profiles initially resembled those of the thalamic or thalamo-cortical lesion regions but then diverged from the LN as clinical recovery progressed. The progressive decline in LN similarity within these regions paralleled rising ADL scores, suggesting that temporary recruitment of non-lesioned regions is a critical, yet time-limited, substrate for functional restitution. Such an alternative biomarker of neural compensation in functional recovery from stroke could be more sensitive than direct measurements of lesion connectivity in the motor network.

Most of the present patients exhibited lesions centered on the thalamus and internal capsule fibers, which are structures fundamental for relaying motor and somatosensory information [4,25,26,27]. Damage to these hubs disrupts integrated sensorimotor loops, hence diminished self-care and mobility (core elements quantified by the ADL scale) are inevitable. Correspondingly, the thalamus was a key region identified in our LN underlying the common sensorimotor dysfunctions suffered by all the patients [28]. The brain appears to counter this network disruption by transiently engaging cortical regions for reinforcing lost inputs or outputs to the damaged relay stations. The identified compensatory regions may facilitate functional recovery by taking over key connections with regions normally connected to the lesions [29,30].

In the early phase after stroke, the left middle frontal gyrus (BA 8, 9) exhibited higher lesion network similarity than in later stages. These dorsolateral prefrontal regions are involved in goal-directed motor planning, monitoring, and working memory—functions that patients may consciously upregulate to compensate for diminished automatic motor control [25,31]. For instance, patients might adopt a more cautious gait, consciously monitoring and adjusting their steps to avoid falls [32]. Similarly, the right lingual gyrus (BA 19), which also temporarily adopted a lesion region-like connectivity pattern [33,34], may enhance visual processing to improve real-time monitoring of limb movement. This is especially relevant given the visual deficits, such as reduced acuity or contrast sensitivity, experienced by some stroke patients.

The right paracentral gyrus (BA 5) contributes to motor control and somatosensory processing of the distal limbs [35]. Damage to sensorimotor cortices and critical efferent/afferent pathways within the internal capsule can cause reduced sensation and fine motor control in the extremities, impairing movement precision and thereby diminishing ADL performance. The observed activity in the right paracentral gyrus (BA 5) may reflect a compensatory mechanism to preserve sensorimotor function and support ADL performance. The right angular gyrus (BA 39), a multimodal association hub, may facilitate adaptation to motor deficits by enhancing sensory feedback or optimizing visuospatial processing to make better use of available sensory cues [27,36]. Finally, the left middle frontal gyrus also showed a decline in functional connectivity similarity over time. Its engagement may help compensate for deficits in memory and cognition during ADL tasks [37].

By definition, compensation is secondary in nature. Because compensatory mechanisms substitute for primary functions, they should diminish as original circuits recover. This pattern aligns with our observation that lesion network similarity decreased alongside ADL improvements. Comparable “rise-and-fall” dynamics have been reported in the contralesional primary motor cortex during arm recovery and in parietal attention networks following neglect therapy [38,39,40].

Compensatory engagement decreased toward the end of the rehabilitation period, by which time more native functional networks had been restored to support ADL. From this perspective, reduced involvement of secondary regions may serve as an early biomarker of functional restoration.

Our study offers novel, network-level mechanistic insight into neural compensation. A key innovation of the lesion network similarity (LNS) approach is its conceptualization of compensation not merely as increased activation in alternative regions, but as the extent to which those regions assume the functional roles of damaged areas within large-scale networks. High similarity early after stroke suggests that regions such as the right angular gyrus are not merely “assisting”—they are reconfigured to process and integrate information in a manner that closely mimics the original, now-disrupted thalamic hub. As recovery proceeds and automaticity is restored, reliance on this effortful, “imitative” processing declines, resulting in reduced similarity. This dynamic, system-level mechanism fundamentally extends and complements simpler models of compensation.

Such a compensatory biomarker may be more sensitive to functional recovery than direct lesion-to-cortex connectivity. In our patients, connectivity changes between the lesion and canonical motor regions were undetectable, whereas changes in lesion network similarity correlated strongly with ADL improvements. This finding is consistent with previous work indicating that network-level plasticity often precedes behavioral gains. For example, Xu et al. reported a correlation between resting-state functional connectivity and motor recovery after subcortical stroke [41]. Golestani et al. performed longitudinal evaluation of resting-state fMRI after acute stroke and also proposed similar points on functional brain plasticity [42].

Despite these insights, several limitations should be noted. First, our model identified only compensatory processes that evolve over time; stationary compensatory mechanisms may have been overlooked. Second, although clinically relevant, reliance on ADL scores may fail to capture plasticity within more specialized functional networks, such as those supporting cognitive or affective functions. Third, the arbitrary matching of one patient to three healthy controls may have influenced the sensitivity of lesion network detection and consequently the identification of compensatory regions. Finally, the use of Pearson correlation alone to assess connectivity similarity leaves open whether alternative metrics (e.g., Euclidean distance or mutual information) might provide additional insights.

Future studies should causally validate the compensatory role of these regions. As suggested, neuromodulation techniques such as transcranial magnetic stimulation (TMS) could be applied to transiently inhibit identified regions (e.g., the right angular gyrus or left middle frontal gyrus) in recovered stroke patients. If these regions are integral to compensation, their disruption should result in a temporary decline in ADL performance. Furthermore, validation in larger independent cohorts and investigation into whether lesion network similarity metrics can predict responses to specific therapies (e.g., TMS) will be crucial for clinical translation.

## 5. Conclusions

This study demonstrates a potential approach for probing neural compensation in stroke through the lens of lesion network similarity. This approach may offer network-based biomarkers for monitoring and evaluating rehabilitation, potentially informing downstream interventions and prognosis. Future research in this direction should examine the utility of LN similarity values derived from different brain regions for predicting outcomes at the beginning of rehabilitation programs.

## Figures and Tables

**Table 1:** Demographic comparison between the stroke patients and their propensity-score-matched healthy controls. * Continuous and discrete variables are compared with group *t*-test and chi-squared tests, respectively.

	Controls (*n* = 15_local_ + 36_remote_)	Patients (*n* = 17)	Statistical *p*-Value *
**Age** **(Y.O. Mean ± SD)**	61.0 ± 14.3	63.2 ± 9.1	*p*_control vs. patient_ = 0.46
**Gender** **(Male | Female)**	32M | 19F	13M | 4F	*p*_control vs. patient_ = 0.38
**Max Head Motion** **(mm, Mean ± SD)**	0.59 ± 0.39	0.79 ± 0.64	*p*_control vs. patient_ = 0.23
**Days From Symptom Onset** **(Mean_i_ ± SD_i_, i = 1, 2, 3)**	NA	2.0 ± 0.9, 104.6 ± 11.7, 210.3 ± 14.2	*p*_Days1 vs. Days2 vs. Days3_ < 0.001
**ADL Scores** **(Mean_i_ ± SD_i_, i = 1, 2, 3)**	NA	32.2 ± 6.3, 75.3 ± 18.2, 88.4 ± 16.8	*p*_ADL1 vs. ADL2 vs. ADL3_ < 0.001

**Table 2:** Significant brain regions detected by the LME analysis of LN similarity. MNI coordinates are center of mass in the orientation of RAI. Specific similarity values at each imaging visit and the effect size of their decreases are also shown.

Region (Brodman Area)	X (mm)	Y (mm)	Z (mm)	Volume (mm^3^)	LN Similarity			Effect Size (η^2^)
					1st	2nd	3rd	
**Right Culmen Lingual Gyrus (BA19)**	−10.1	54.6	−0.6	594	0.76	0.48	0.41	0.13
**Right Angular Gyrus (BA39)**	−45.6	65.6	29.6	540	0.73	0.49	0.37	0.17
**Right Paracentral Gyrus (BA5)**	−10.6	37.8	50.2	513	0.88	0.70	0.62	0.10
**Left Middle Frontal Gyrus1 (BA9)**	49.7	−23.5	37.9	405	0.69	0.47	0.33	0.20
**Left Middle Frontal Gyrus2 (BA8)**	27.0	−26.3	43.3	405	0.68	0.49	0.39	0.14

## Data Availability

No new data were created or analyzed in this study.
